# Development and Validation of the Self-Acceptance Scale for Persons with Early Blindness: The SAS-EB

**DOI:** 10.1371/journal.pone.0106848

**Published:** 2014-09-30

**Authors:** Fabiane Frota da Rocha Morgado, Angela Nogueira Neves Betanho Campana, Maria da Consolação Gomes Cunha Fernandes Tavares

**Affiliations:** 1 Department of Physical Education, University of Campinas, Campinas, São Paulo, Brazil; 2 Sagrado Coração University, Bauru, São Paulo, Brazil; National Laboratory of Pattern Recognition, China

## Abstract

Investigations of self-acceptance are critical to understanding the development and maintenance of psychological health. However, valid and reliable instruments for measuring self-acceptance in persons with early blindness have yet to be developed. The current research describes three studies designed to develop and validate the Self-acceptance Scale for Persons with Early Blindness (SAS-EB). In Study 1, we developed the initial item pool. Thirty-three items were generated, based on data from specialized literature and from 2 focus groups. Items were organized in a three-factor structure, theoretically predicted for SAS-EB - (1) body acceptance, (2) self-protection from social stigmas, and (3) feeling and believing in one's capacities. In Study 2, information obtained from a panel of 9 experts and 22 persons with early blindness representing the target population was used to refine the initial item pool, generating a new pool of 27 items. In Study 3, 318 persons with early blindness (141 women and 177 men), between 18 and 60 years of age (M = 37.74 years, SD = 12.37) answered the new pool of 27 items. After the elimination of 9 items using confirmatory factor analysis, we confirmed the theoretical three-factor structure of the SAS-EB. Study 3 also provided support for the scale's internal consistency and construct validity. Finally, the psychometric properties of the SAS-EB, its utility, and its limitations are discussed along with considerations for future research.

## Introduction

Self-acceptance is an important concept in understanding the development of psychological health [1-2-3]. It is defined as an individual's acceptance of all of his/her attributes, positive or negative. Self-acceptance enables an individual to appropriately evaluate his/her efficient and inefficient features and accept any negative aspects as parts of their personality [4]-[5].

Three main attitudes are markedly inherent in self-acceptance. The first is “*body acceptance*”, defined by Tilka [6] (p. 59) as “expressing comfort with and love for the body, despite not being completely satisfied with all aspects of the body”. Another important attitude is “*self-protection from negative judgments from others*”, which consists in a lack of concern that others are judging oneself negatively [7]-[8]. The third attitude focuses on “*feeling and believing in one*'*s capacities*”, which includes recognizing, appreciating and developing positives thoughts and feelings about one's capacities and realizations [9]-[10].

Self-acceptance has been positively associated with different positive aspects of mental health, such as high self-esteem, interpersonal satisfaction and affect regulation [1]-[11]. Given its features, self-acceptance allows the individual to experience a healthy relationship with the self, contributing to the development of a positive body image [12-13-14]. In contrast, self-acceptance has been negatively associated with different psychopathologies, such as depression and anxiety [15]-[16]. Additionally, a lack of self-acceptance is considered as a principal contributor to the development and maintenance of eating disorders and obesity [17]-[18] and may contribute to the emergence of a negative body image [13].

In individuals with a disability, self-acceptance has been hypothesized to be a particularly important construct. It is considered to be a crucial psychological advantage, especially because it enables individuals to view themselves positively, to accept limitations stemming from the disability, and to be better equipped to cope with the disability and to establish healthy practices [19]. Lack of self-acceptance is characterized by feelings of worthlessness, inadequacy, depression, self-blame and self-hatred, which block motivation, inhibit positive behaviors, and cause difficulties in rehabilitation and adjustment [20]-[21]. Despite the importance of this concept, no measurement scales are available to assess self-acceptance in people with early blindness.

Early visual deprivation may hinder emotional and psychological development, as well as personality organization. The psychological adjustment of these individuals in a seeing world may be more egocentric, especially if their environment is deficient in interpersonal interaction, which contributes to the complexities of personality development, impacting self-acceptance [22]-[23]. In addition, reports have been published on the presence of eating disorders in persons with early onset blindness [24-25-26-27-28-29-30-31]. A possible explanation for the development of these disorders may be the presence of low levels of self-acceptance. However, so far, this has not been well explored.

The Self-Acceptance Questionnaire (SAQ) [32] is the only available instrument specifically adapted for individuals in China to assess self-acceptance in individuals with visual impairment. Although the psychometric properties of the SAQ are adequate, this instrument is not specifically suited to persons with early blindness. The lack of an appropriate scale to assess self-acceptance in people with early blindness limits the advancement of studies in this field, hampering the development of efficient interventions for prevention and therapy. Measuring self-acceptance for sample groups of individuals with early blindness is important in order to help identify people at risk for a lack of self-acceptance. Thus, there is a great need for the development and validation of a specific scale for these persons.

The aim of this study was to create and evaluate the psychometric properties of the new Self-acceptance Scale for Persons with Early Blindness (SAS-EB). Building on the large body of research on scale development [33-34-35-36], the present paper describes three studies: (1) item generation, (2) scale refinement, and (3) confirmatory factor analysis, reliability and construct validity. This study was approved by an institutional review board (Research Ethics Committee of the Campinas State University - Rep. 187/2010).

## Study 1: Item Generation

The purpose of Study 1 was to develop the SAS-EB items. The initial item pool generation was created and selected on the basis of two information sources: data acquired from the specialized literature and from focus groups including participants with early blindness. Consistent with previous theory and research on self-acceptance [6-7-8-10], we hypothesized that the SAS-EB would consist of three factors: (1) body acceptance, (2) self-protection from negative judgments from others, and (3) feeling and believing in one's capacities.

### Methods

#### Participants

There were 11 participants with early-onset blindness (5 men and 6 women, mean age  = 34.8, SD = 7.3 years, range 26–48 years) conveniently recruited from the Benjamin Constant Institute, located in Rio de Janeiro, Brazil. We considered blind subjects, participants who were self-reported to have vision scores of less than 3/60 in their best eye, with the best optical correction, i.e., those who are able to see only 3 meters ahead, when a person with normal vision could see up to 60 meters [37]. Early blindness occurs in individuals who had lost their vision in early childhood (range: birth-8 years of age). The present definition of early blindness is consistent with a number of prior studies [38-39-40], but differs from some, which consider early blindness to be loss of vision before the age of 1 [41] or between the ages of 1.4–13 years [42]. In order to be eligible to participate in this study, participants must: (1) have reported a diagnosis of early blindness, with no other clinical conditions, (2) be ≥18 years of age, and (3) have agreed to freely participate in the research.

#### Procedure

Two focus groups were conducted. The first included six women, while the second group consisted of five men. Men and women were separated in order to achieve greater homogeneity among the participants. Prior to the group formation, information about the purpose of the study and confidentiality were provided and informed written consent was obtained. No compensation was offered.

Each focus group session lasted 90–120 min and was led by two moderators with prior experience in conducting focus group. Moderators sat around an oval table with the participants, one on each side of the table. The main moderator led the discussion and the second moderator intermittently introduced questions of interest regarding the understanding of self-acceptance.

Moderators asked different questions related to self-acceptance, structured on the three factors we hypothesized to constitute the construct. As examples of moderate issues, we proposed: a) to the first factor - Comment on the relationship that you establish with your body – is it positive, is it negative, is it irrelevant? Does the blindness interfere with your relationship to your own body? Do you like to take care of yourself? How is the care of the body in the absence of vision? b) to the second factor - How does society view a person who cannot see? Is the opinion of others important to you? Does the opinion of others interfere with your feelings about yourself? c) to the third factor - What is needed for you to develop and maintain feelings and beliefs of capability? What would increase your love for yourself? Are the opinions of people who are blind valued? These questions were previously established. However, during the focus group, other questions emerged and the moderators had the freedom to go beyond those previously established. The participants gave their opinions of the issues, disagreed amongst themselves, agreed, discussed controversial issues, and, in short, intensely participated in the discussions. Moderators avoided giving any opinion, leading the group so as not to interfere with the view of the participants, thus avoiding bias.

The sessions also had an observer, who made important notes about the attitudes of the participants, as well as a qualified professional who audio/video-recorded the discussions, with the participants' permission. The discussion material was literally transcribed. Qualitative data from the focus group were analyzed thematically with the content analysis [43].

### Results

The first study provided the initial item pool generation. There were 33 items generated from the specialized literature and from two focus groups. Items were rated on a 5-point Likert scale (1 =  *never*, 2 =  *seldom*, 3 =  *sometimes*, 4 =  *often*, 5 =  *always*) and were distributed in the three initially hypothesized factors (Table S1 in File S1).

The first factor, labeled *body acceptance*, was central to the participants' conceptualization of self-acceptance. Congruent with a previous study [44], participants in this study reported that, even without visual experience since an early age, satisfaction with appearance, body care, body love, and the value attributed to one's body are inherent attributes of self-acceptance. Thus, 14 items were generated to compose this factor and assess the expression of love, care and satisfaction with the body.

The second factor was changed. In our initial hypothesis, it had been labeled *self-protection from negative judgments from others*, which consisted in evaluating the ability of the individual to accept themselves without the worry of being negatively evaluated by others. However, after an analysis of the discussions from the two focus groups and in accordance with studies of Brittain [45], Hughes [46], Lobianco and Sheppard-Jones [47], Sahin and Akyol [48], Tripp [49], and Watson [50], we found that for individuals with disabilities, an important source of negative judgment comes from social stigmas. Stigma is an attribute that others perceive to be deeply discrediting [51]-[52]. In disabled persons, stigma usually demonstrates discrimination, avoidance, social exclusion [49], invalidation, and disfigurement [46]. Stigmas associated with disability may have a negative impact on self-acceptance [20]. Therefore, the second factor is now termed *self-protection from social stigmas*, and comprises 5 items, which assess the subjects' ability to accept themselves without worry about negative judgments from others related to social stigma.

The third factor, labeled *feeling and believing in one*'*s capacities*, assesses the positive thoughts and feelings about the capacities of the individual related to daily life with early blindness. These thoughts and feelings were considered critical to the development and maintenance of self-acceptance in the participants. This factor is composed of 14 items. We hypothesized that all of the individual items from all three categories would be capable assessors of the self-acceptance of persons with early blindness.

As a result, we constructed an initial version of the SAS-EB, which will be subsequently refined over the course of following studies.

## Study 2: Scale Refinement

The aim of Study 2 was to assess the theoretical quality of the first version of the SAS-EB. We assessed the content validity of the new scale by consulting both a panel of experts and persons with early blindness representing the target population.

### Methods

#### Participants

A panel of nine independent Ph.D. - level scientists with expertise in self-acceptance, visual impairment or scale development research reviewed the preliminary version of the SAS-EB. This panel included three self-acceptance specialists, three professors who had experience in research on visual impairment, one professor who had experience in research on both visual impairment and self-acceptance, and two experts who had published several scale development-related articles.

A group of 22 participants with early blindness, 10 women and 12 men, aged 29 to 48 years (*M* = 34.2, *SD* = 7.2), recruited from the Benjamin Constant Institute and from the Association for the Blind of Juiz de Fora, Minas Gerais, Brazil, with varying levels of education (range: elementary - higher education) and different social traits (living in the boarding system in the Association for the Blind of Juiz de Fora (*n* = 14), Braille teacher (*n* = 4), and Braille reviewer (*n* = 4) of the Benjamin Constant Institute), agreed to assess the preliminary version of the SAS-EB, in the “pretest” procedure [35]. The criteria for participant inclusion were: (1) diagnosis of early blindness, with no other clinical conditions, (2) aged ≥18 years, and (3) agreed to freely participate in the research.

#### Instruments

The **Content Validity Form** was a specific judgment report created especially for this purpose. We asked the participants to judge how relevant they thought the content of each item was to assess self-acceptance. They might rate relevance to be high (+1  =  appropriate item), moderate (0  =  rewrite item), or low (-1  =  delete item). In addition, we invited the experts to comment on individual items as they saw fit, and to make notation of new items that they would recommend, helping us to maximize the content validity of the SAS-EB.

#### Procedures

All participants provided written informed consent to participate in the study. No compensation was offered. The participants assessed item relevance, clarity, conciseness, and redundancy [33]. The ratio of content validity criterion was 0.70^36^. Items considered to be of low relevance for at least 70% of the judges were eliminated. Items considered to be of moderate relevance for at least 70% of the judges were rewritten. Items considered problematic regarding these aspects by at least 70% of the experts were eliminated and those considered partly problematic by at least 70% of the experts were rewritten.

After the expert review, a second and adjusted version of the scale was submitted to the pretest. Three pretests were performed. In the first, seven subjects with early blindness were individually invited to participate in the study and all agreed to participate. Then, in a reserved and silent room, the researcher read the scale for each participant, individually, asking him/her to answer items by “thinking aloud”. At the same time, according to the responses of the participant, the researcher completed a judgment report that had been specially developed for this stage of the study. In this report, we evaluated: (1) the understanding of item content by the participants, and (2) the level of difficulty in answering the items. All questions asked of the participants in relation to item content and the difficulty in answering the questions were recorded for later analysis. In the second pretest, seven other subjects with early blindness were invited to participate in the study and all gave their consent. The procedures were the same as in the first pretest; however, a new judgment measure was used to assess the reformulated scale from the first pretest. In this new report, we also evaluated: (1) the understanding of the content of the items by the participants, and (2) the level of difficulty in answering the questions. Finally, in the last pretest, eight other representatives of the target population agreed to participate in the study. This pretest was different from the previously administered test. We evaluated whether or not the aural application of the scale, appropriately reproduced via electronic equipment, would be effectively understood. The aurally applied scale could facilitate and homogenize the data collection procedure. Then, beyond the researcher reading the scale to the participants, a previously recorded, standardized reading could be used for all study subjects. Participants were asked which method between the two (reading or audio) they felt more comfortable answering. The answers were recorded for later analysis. Data analysis of the pretest was given qualitatively, i.e., by the subjective evaluation of the researcher.

### Results

Study 2 provided the SAS-EB content validity through the opinions of both experts and representatives of the target population. The experts review resulted in the rewording of 21 items, the deletion of 7 items that were considered of low relevance in content or were redundant, and the inclusion of 1 item, leaving a total of 27 items (*body acceptance*  =  11 items, *self-protection from social stigmas*  =  4 items, and *feeling and believing in one*'*s capacities*  =  12 items). These 27 items formed the second and adjusted version of the SAS-EB, which was used in the subsequent pretest.

The first pretest indicated that 6 items were difficult for the participants to understand. As a result, the content of these items was rewritten, and a third and refined version of the SAS-EB was directed for a second pretest. In the second pretest, the participants were able to understand all of the item content and they had no difficulty answering the questions from the scale. In the last pretest, the third and refined version of scale (Table S2 in File S1) was applied with an audio CD and received the approval of the majority (*n* = 7) of participants. Therefore, this scale was aurally applied in the following procedures.

## Study 3: Confirmatory Factor Analyses, Reliability and Construct Validity

The main goals of Study 3 were to perform Confirmatory Factor Analyses (CFA) to test the three-factor model of the SAS-EB that was hypothesized in Study 1 and refined in Study 2 and to assess the SAS-EB internal reliability and construct validity.

In relation to convergent validity, we hypothesized that, consistent with previous theory and research about self-acceptance [5-20-53-54-55-56-57], the SAS-EB factors would be positively related to certain components, e.g., body satisfaction, visual impairment acceptance, level of education, and intensity of physical activity. Conversely, they would be negatively related to both intensity of perceived social stigma and BMI. These relationships were examined in the present study.

Concerning discriminant validity, research suggests that self-acceptance may encompass several different distinct criteria groups. For example, professional status includes variables for being in or out of the job market [55]-[58], marital status can be with or without a romantic partner [59], the sex variable encompasses men and women [5]-[60], age can be young or old [5], and BMI variables include normal weight, overweight or underweight [57]. Based on this evidence, we also hypothesized that the SAS-EB could differ significantly with varying group criteria.

### Methods

#### Participants

In Study 3, 339 individuals with early blindness were invited. Twenty-one individuals refused to participate (12 male, 9 female, 25 to 35 years old). The new sample included 318 individuals (141 women and 177 men), between 18 and 60 years of age (*M* = 37.74 years, *SD* = 12.37). The average self-reported weight was 69.14 kg (*SD* = 13.80 kg), height was 1.62 m (*SD* = .09 m), and average self-reported body mass index (BMI) was 26.05 kg/m^2^ (*SD* = 4.5). Our sample size exceeded the number of participants needed (270) for a case-to-parameter ratio of 10:1, in order to examine the model fit and to conduct all planned analyses [33]-[34].

The most common cause of early blindness was congenital glaucoma (*n* = 99, 31.1%), followed by undetermined causes (*n* = 46, 14.5%), retinitis pigmentosa (*n* = 27, 8.5%), retinopathy of prematurity (*n* = 26, 8.2%), optic nerve atrophy (*n* = 23, 7.2%), congenital cataract (*n* = 17, 5.3%) and other (*n* = 80, 25.2%). Most of the participants described themselves as single (*n* = 118, 37.1%), employed (*n* = 170, 53.5%), having completed a basic education (*n* = 104, 32.7%) and as not routinely engaging in exercise (*n* = 203, 63.8%).

Participants were recruited from three distinct regions of Brazil: Midwest, Southeast and Northeast. Selection of participants occurred both in centers specializing in the care of people with visual impairments, including educational institutions and public philanthropic institutions, and at cultural and scientific events in which the primary audience was composed of people with visual impairments - Blind Encounter Brazil (Rio de Janeiro and Fortaleza) and the XV Brazilian Meeting of DOSVOX users (Rio de Janeiro).

#### Instruments

##### Self-acceptance Scale for Persons with Early Blindness (SAS-EB)

The scale used was the same developed in Study 1 and refined in Study 2.

##### Demographic questionnaire

The questionnaire was developed specifically for this study. Participants were asked about their age, weight, height, level of education, intensity of physical activity, professional status, and marital status. They were also asked about their level of body satisfaction (*Regarding your body, you are: (1) dissatisfied, (2) somewhat satisfied, (3) indifferent, (4) very satisfied, (5) completely satisfied*), visual impairment acceptance (*Do you accept your blind condition? The response options were: (1) never, (2) seldom, (3) sometimes, (4) often, and (5) always)*, and intensity of perceived social stigma (*When you go out into the street, at work, at school, or in your family, have you experienced any kind of social stigma regarding your blindness? The answer options were rated on a 5-point scale ranging from (1) never to (5) always*).

#### Procedure

The researcher individually invited all participants. We included participants who self-reported early blindness, with no other clinical conditions, who freely agreed to participate in the study, and who were between 18 and 60 years old. Participants who: (1) did not precisely report the age at which they became blind, (2) did not precisely declare their visual condition (low vision or blindness), (3) reported having acquired blindness after 8 years of age, or (4) reported having low vision were excluded. All participants included in the study (n = 318) were informed about the research procedures and provided written informed consent. No compensation was offered.

After agreeing to participate in the study, the volunteer was individually led to a quiet, reserved room. The scale instructions were then played on an audio recording. The researcher took note of all answers for later analysis. Each volunteer took approximately 20 minutes to answer the survey. The LISREL system [61] and SPSS software were used for statistical analyses.

### Results

#### Confirmatory Factor Analyses

Using the LISREL system, we conducted the CFA on the 27 item responses testing for the hypothesized three-factor model of SAS-EB. The unweighted least square method of estimation was used because of the lack of multivariate normality of our data. The *listwise* deletion criterion was adopted for missing data. GFI (Goodness-of-fit)>.90, AGFI (Adjusted Goodness-of-Fit)>.90, NNFI (Non-normed Fit)>.90, CFI (Comparative Fit)>.90, X^2^/df (Standardized Chi-square) <3, and RMSEA (Root Mean Square Error of Approximation) <.08 indices evaluated the model fit. Items were considered for deletion if they produced scores that had factor loadings ≤.35 [36].

As result, the hypothesized three-factor model did not provide an adequate fit to the data (χ^2^/df = 2.21, RMSEA = .062, GFI = .94, AGFI = .93, NNFI = .96, CFI = .96). The GFI and AGFI statistics were below (<.95 for both) cutoffs for good fit. Items 11 (λ = .24) and 21 (λ = .21) had low factor loading and were eliminated. Once these items were eliminated, the model provided a good fit to the data (χ^2^/df = 2.13, RMSEA = .060, GFI = .96, AGFI = .95, NNFI = .97, CFI = .98). However, items 2, 7, 8, 25 and 27 had the lowest factor loading (λ = .34, λ = .31, λ = .35, λ = .33, and λ = .35, respectively) and showed large associated residuals (.88,.89,.87,.87,.85, respectively), therefore, we consequently eliminated them. After this elimination, we decided to also eliminate items 4 and 18 because of their high positive residuals (≥±2.58). Finally, a better model fit was achieved (χ^2^/df = 1.86, RMSEA = .052, GFI = .97, AGFI = .96, NNFI = .98, CFI = .99). Subsequently, after the elimination of the nine items, we confirmed the factor structure of the SAS-EB three-factor model. The SAS-EB now has 18 items: first factor  = 6 items, second factor  = 4 items and third factor  =  8 items. Items 9, 10, 13, 16, 20, 22, 24, and 26 had reverse scores (Table 1).

**Table 1:** Item-factor loadings and descriptive statistics of SAS-EB item scores in Study 3.

Items		M	SD	λ	θ
	**First Factor: Body acceptance**				
BA1	Do you like your body the way it is? [*Você gosta de seu corpo como ele é?*]	4.06	1.14	.63	.59
BA3	Do you like your appearance, for example, your hair, your face, the way you dress? [*Você gosta de sua aparência, por exemplo, seu cabelo, seu rosto, seu modo de vestir?*]	4.23	.95	.54	.70
BA14	Do you like yourself the way you are? [*Você gosta do jeito que você é?*]	4.31	.93	.76	.41
BA15	Do you take care of your appearance, for instance, your hair, your skin, and your clothes? [*Você cuida de sua aparência, por exemplo, de seu cabelo, sua pele, seu vestuário?*]	4.44	.81	.40	.84
BA17	Do you recognize your good qualities? [*Você reconhece suas qualidades?*]	4.19	.92	.39	.84
BA19	Do you think you are physically attractive? [*Você se considera fisicamente atraente?*]	3.29	1.12	.46	.78
	**Second Factor: Self-protection from social stigmas**				
SP13	Do you worry about negative attitudes from society regarding your blindness? [*Você se incomoda com algumas atitudes negativas da sociedade a respeito de sua condição de cego?*]	3.34	1.31	.46	.78
SP20	Do you get annoyed with prejudiced opinions of your blindness? [*Você se incomoda com opiniões preconceituosas a respeito de sua cegueira?*]	2.84	1.45	.52	.72
SP22	When you observe some kind of prejudice related with your blindness, do you feel yourself to be less of a person? [*Quando você observa algum tipo de preconceito social em relação a sua cegueira, você se sente inferior às outras pessoas?*]	2.02	1.23	.72	.48
SP26	Do you worry about preposterous questions about your blindness? [*Você se incomoda com perguntas inadequadas sobre sua cegueira?*]	2.22	1.36	.43	.81
	**Third Factor: Feeling and believing in one**'**s capacities**				
FB5	Do you think that you are capable of deciding what is best for you? [*Você se considera capaz de decidir o que é melhor para você?*]	4.50	.76	.43	.81
FB6	Are you highly positive about your life? [*Você é muito positivo em relação a sua vida?*]	4.31	.87	.54	.54
FB9	Does blindness cause difficulties in your social interactions? [*Ser cego lhe dificulta se relacionar com outras pessoas?*]	2.35	1.2	.51	.73
FB10	Does blindness hinder you from taking part in your favorite activities? [*Sua cegueira lhe atrapalha a fazer coisas que você gosta?*]	2.33	1.07	.54	.71
FB12	Do you feel that you are capable of overcoming your day-to-day difficulties? [*Você se sente capaz de superar as dificuldades que possam existir no seu dia-a-dia?*]	4.32	.83	.57	.66
FB16	Does being blind have negative effect on your romantic relationships? [*Ser cego lhe dificulta ter um relacionamento amoroso?*]	2.05	1.26	.55	.69
FB23	Are your opinions highly respected in the places you give them, for example, in your work, school, and home? [*Suas opiniões são muito respeitadas nos lugares que você frequenta, por exemplo, trabalho, escola, lar?*]	3.61	.94	.36	.87
FB24	Does blindness hinder you from doing the things you have to do? [*Sua cegueira lhe atrapalha a fazer coisas que você necessita fazer?*]	2.48	.99	.55	.69

**Note**. BA = body acceptance, SP = self-protection from social stigmas, FB = feeling and believing in one's capacities, M = mean, SD =  standard deviation, λ = item-factor loading, θ = error term.

Brazilian Portuguese original version of the items are given in brackets.

#### Reliability and Construct validity

To determine the internal consistency of the SAS-EB scores, we used Cronbach's alpha and examined item total and inter-item correlations. Alpha values greater than or equal to.6 suggest acceptable reliability for exploratory studies [34]. Item-total correlations between.30 and.70 and a mean inter-item correlation greater than.20 indicate adequate reliability [34]-[62]. In this study, all three factors showed moderate reliability indices: the first factor (α = .70), second factor (α = .69), and third factor (α = .74). Item-total correlations ranged between.31 and.62 and mean inter-item correlation ranged between.28 and.26, providing additional evidence of internal reliability (Table 2).

**Table 2:** Item total and mean interitem correlations, alphas, and Spearman's correlations among the measures of Study 3 (N = 318).

Factors	Items	Item-total (r)	Mean interitem (r)	α	Body satisfaction (rho)	Visual impairment acceptance (rho)	LevelEduc. (rho)	Intensity physical activity (rho)	Intensity perceived social stigma(rho)	BMI(rho)
	BA1	.48								
	BA3	.52								
**Body**	BA14	.62	.28	.70	.52**	.33**	−.01	.18	−.63	−.03
**acceptance**	BA15	.31								
	BA17	.34								
	BA19	.42								
**Self-**	SP13	.51								
**protection**	SP20	.54								
**from social**	SP22	.41	.36	.69	.21**	.25**	.06	.18	−.15**	−.02
**stigmas**	SP26	.42								
	FB5	.34								
	FB6	.42								
**Feeling and**	FB9	.42								
**believing in**	FB10	.5	.27	.74	.32**	.32**	.13*	.24**	−.14*	.01
**oneself**	FB12	.47								
**capacities**	FB16	.54								
	FB23	.32								
	FB24	.47								

** Correlation is significant at.01 (2-tailed)

* Correlation is significant at.05 (2-tailed)

P<0,001

rho  =  Spearman's correlation.

To determine convergent validity, we used Spearman's correlations between each of the three factors of the SAS-EB and body satisfaction, visual impairment acceptance, level of education, intensity of physical activity, intensity of perceived social stigma, and BMI variables (Table 2). As hypothesized, higher scores of the SAS-EB were associated with higher body satisfaction: first factor (*r_s_* = .52, *p*<.01), second factor (*r_s_* = .21, *p*<.01), and third factor (*r_s_* = .32, *p*<.01). For visual impairment acceptance, our results were also as predicted. We found positive and significant associations for the first factor (*r_s_* = .33, *p*<.01), second factor (*r_s_* = .25, *p*<.01), and third factor (*r_s_* = .32, *p*<.01). Additional evidence of convergent validity was shown for the second and third factors. The second factor showed a significant, negative correlation with intensity of perceived social stigma (*r_s_* = −.15, *p*<.01), confirming our previous hypotheses. The third factor also revealed a significant, negative correlation with intensity of perceived social stigma (*r_s_* = −.14, *p*<.05), and a significant, positive correlation with level of education (*r_s_* = .13, *p*<.05), and intensity of physical activity (*r_s_* = .245, *p*<.01). Contrary to what we initially hypothesized, BMI did not correlate with any of the factors. However, the correlations that were found indicate satisfactory parameters of convergent validity for the three factors of SAS-EB.

To determine discriminant validity, the Mann-Whitney test was conducted to evaluate differences in SAS-EB factor scores concerning different group criteria, e.g., working (*n* = 170) and unemployed (*n* = 148), with a romantic partner (*n* = 164) and without a romantic partner (*n* = 154), men (*n* = 177) and women (*n* = 141), younger, 18–39 years old, (*n* = 176) and older, 40–60 years old, (*n* = 142), and normal weight (*n* = 126) and overweight or underweight (*n* = 192). Our results showed discriminant validity for the second and third factor. The *self-protection from social stigmas* factor significantly discriminated groups with and without a romantic partner (participants with a partner had higher mean ranks than those without partner, p<.05), and men and women (men had higher mean ranks than women, p = .001). The *feeling and believing in one*'*s capacities* factor significantly discriminated groups by employment status (subjects in the job market had higher mean ranks than subjects outside, p<.05) and by age (younger participants had higher mean ranks than those who were older, p = .05). No factor discriminated between BMI groups, contradicting our previous hypotheses. The second and third factors showed evidence of discriminant validity.

## General Discussion

The present study was designed to develop a reliable and valid measure of self-acceptance for use with samples from Brazil with early blindness. Results of the three studies suggest that the SAS-EB is reliable and valid for measuring self-acceptance in this portion of the population. In Study 1, we initially developed the three-factor self-acceptance model (*body acceptance*, *self-protection from social stigmas,* and *feeling and believing in one*'*s capacities).* The initial item pool included 33 items carefully drawn to be easily understood by the target population and to represent the hypothesized three factors. The first factor was created to assess the level of love, care and satisfaction with oneself, including one's own body and bodily appearance. The second factor was structured to assess the organization of the individuals' protection cognitive filter, qualifying them to reject negative information about their blindness derived from social stigmas. The third factor was organized to assess the feelings and beliefs of one's capacities to face everyday situations identified by participants as important to the development and maintenance of self-acceptance.

In Study 2, we confirmed the content validity of the SAS-EB. After a review by nine experts in self-acceptance, visual impairment, or scale development research, a total of 27 items were retained from the initial pool of 33 items. As a result of the three pretests given to 22 representatives of the target population, minor adjustments were made in the wording of 6 items. One important aspect of this study was that it tested and approved the application of the new scale in an audio format, adapting it specifically to our sample with blindness. The audio version of the SAS-EB is an improvement over the previous study [32], because it makes the instrument easier to administer to large sample groups of blind individuals, making homogeneous conditions of application and data collection feasible.

In Study 3, a psychometric investigation of the SAS-EB indicated that it shows satisfactory evidence of factorial validity, internal reliability and construct validity. Results of the CFA revealed that the data fit the hypothesized three-factor model well, with item scores showing suitable loadings (range λ = .36 – λ = .76) on the intended factors. However, to better fit the model, it was necessary to reduce the number of items, retaining 18 of the initial 27 initial. Despite the brevity of the scale, the three factors were comprised of a number of indicators that exceeded the minimum number required of three item scales [36]. Moreover, the SAS-EB three-factor model with 18 items provided a quick and comprehensive assessment of self-acceptance in persons with early blindness.

The internal reliability of SAS-EB was moderate (range α = .69–.74). This result is close to that reported in a previous study on scale development of self-acceptance with sighted people (α = .72) [4], but it differs from other similar previous investigations (α = .42 [63]; α = .85 [14]; α = .83 [32]). The modest alpha coefficients found in this study likely reflect the small number of indicators per factor and the fact that items were chosen to represent a multifaceted construct, such as self-acceptance. Although moderate, alpha values seem acceptable given that the scale presented appropriate values of item total and mean inter-item correlation, supporting the internal consistency of the scale.

In relation to convergent validity, the results indicated that the greater self-acceptance was related to higher levels of body satisfaction and visual impairment acceptance, confirming previous research [20-53-54]. Further evidence of convergent validity was also found. Consistent with prior research examining the impact of perceived social stigma on psychological correlates of obese Americans [57], the *self-protection from social stigmas* factor correlated modestly and negatively with the intensity of perceived social stigma, showing that the perception of social stigmas has a deleterious effect on the self-protection of people with early blindness. Similarly, the *feeling and believing in one*'*s capacities* factor correlated modestly and negatively with the intensity of perceived social stigma, clarifying that the perception of social stigmas also has a negative effect on the feelings and beliefs of self-capacities in individuals with early blindness. In addition, greater scores for *feeling and believing in one*'*s capacities* were modestly related to higher levels of education, as found by Balon *et al*. [55], and intensity of physical activity, as found by Crone, Smith and Gough [56], highlighting that both education about and practice of regular physical activity are important elements to increase levels of self-acceptance in persons who are blind from an early age. Taken together, these findings confirm the construct validity of the SAS-EB.

When investigating discriminant validity, this study showed satisfactory results for *self-protection from social stigmas* and *feeling and believing in one*'*s capacities*, but not for the *body acceptance* factor. Consistent with prior research, the second factor significantly discriminated criteria groups related to marital status [59] and to sex [5]-[60]. The third factor significantly discriminated criteria groups related to professional status [55]-[58] and age [5]. Given that these criteria groups should theoretically discriminate, this study provided support to confirm the discriminant validity of the second and third factors of the SAS-EB. A possible explanation for the lack of discriminant validity for the first factor may be the fact that specific criteria groups related to *body acceptance*, such as internalization of the thin ideal and self-esteem [64], were not evaluated. Future research could investigate the ability of the *body acceptance* factor to successfully differentiate between the two groups mentioned. However, the biggest challenge would be to find appropriate ways to evaluate these variables in the population of the visually impaired.

Carr and Friedman [57] highlighted that higher BMI scores are associated with lower levels of self-acceptance. Accordingly, a significant negative correlation was expected between BMI and factors of the SAS-EB and that the factors of the new scale would be able to differentiate between groups with different measures of BMI. Curiously, contradicting our previously formed hypothesis, the findings found no convergent or discriminant validity regarding the measure of BMI. Two hypotheses may be formulated to explain this finding. The first is that BMI was self-reported, which may have affected the quality of collected measurements of weight and height. In accordance with Gardner, Jappe and Gardner [65], self-reporting weight and height can be a problem, because individuals often overestimate height and underestimate weight, making this measure somewhat inaccurate. In addition, numerous variables affect self-reporting, including sex, age, race/ethnicity, marital status, income, and activity level. The second and more probable hypothesis is that self-acceptance for individuals with early blindness would be independent of body size and weight. It would have to be an unconditional acceptance of oneself, allowing individuals who are blind from an early age to develop and maintain high levels of self-acceptance, even when their appearance does not conform to societal ideals of attractiveness. However, future work in this area should use real measurements of weight and height, examining the relationship between the SAS-EB and BMI.

The present scale offers advantage over the only existing scale for the assessment of self-acceptance in individuals with visual impairment (SAQ), particularly, because it was created and validated specifically for people who were blind at an early age (birth-8 years of age), respecting and appraising the peculiar emotional and psychological characteristics of this population. However, it is worth highlighting that the age limits for the establishment of early blindness are controversial in the literature and this fact can hinder the wide application of the SAS-EB. In this sense, we suggest future studies that investigate the validity of the SAS-EB in groups of blind subjects of different ages, making it possible to know if the new scale is associated with the age of the onset of blindness.

Despite its contributions, the limitations of this study also need to be addressed. The first relates to self-reported weight and height used in study 3. The inaccuracy of self-estimates and inaccuracy in individual perception may have affected the validity of the results, since reported BMI does not allow for the confirmation of convergent or discriminant validity. However, some authors have defended the use of self-reported weight and height as actual predictor of these measures, for the reason that the discrepancies between actual and self-reported measure were sufficiently small to allow using self-estimates in place of measurements [65]-[66]. In addition, Study 3 recruited a peculiar population - numerous and hard to reach - marking it difficult to achieve ideal conditions for data collection (access to laboratories with scale and stadiometer), requiring that the measurements of weight and height were quick, thus self-reported. Future studies could examine these factors.

The second limitation was the lack of the use of a validated scale to assess the main variable used to check the convergent validity for body satisfaction. Although there is a valid and reliable Brazilian measure for body satisfaction in blind people (Three-Dimensional Body Rating Scale (3BRS) [67]), it was validated for congenitally blind women, and would not be suitable for men or for people who became blind after the age of five, as in the case of some members of this sample. Similarly, another limitation was that no validated scale was used to assess the acceptance of the visual impairment variable for convergent validity. However, in this instance, no specific Brazilian scale was found for this type of measure.

Finally, one additional limitation relates to the inability of scale to provide information on normal or abnormal levels of self-acceptance, failing to derive a standard for Brazil. As a result, this study is limited in its application. Therefore, new and more sophisticated analyses should be performed to establish the norm for people with early blindness in the Brazil.

Despite these limitations, the evidence presented in this preliminary and exploratory study suggests that the SAS-EB may serve as a useful tool in future research related to self-acceptance in persons with early blindness in Brazil. In general, results from the three studies demonstrated that the SAS-EB produced scores that were reliable, with evidence supporting multiple facets of construct validity for the factors intended to measure *body acceptance*, *self-protection from social stigmas,* and *feeling and believing in one*'*s capacities.* However, future research is needed to provide additional evidence regarding the psychometric properties of the SAS-EB.

## Supporting Information

File S1 **Supporting tables. Table S1, The initial item pool (thirty-three items) generated in Study 1. Table S2, The item pool refined in Study 2 after content validity (panel of experts and pretest).**
(DOCX)
