# Supplementary material for: Development and Validation of the Self-Acceptance Scale for Persons with Early Blindness: The SAS-EB
Source: PLoS One. 2014 Sep 30;9(9):e106848. doi: 10.1371/journal.pone.0106848 (PMC4182093; doi:10.1371/journal.pone.0106848)
Supplement: File S1 — Supporting tables. Table S1, The initial item pool (thirty-three items) generated in Study 1. Table S2, The item pool refined in Study 2 after content validity (panel of experts and pretest). (DOCX) [file pone.0106848.s001.docx]

**Table S1 -** The initial item pool (thirty-three items) generated in Study 1

| **Items** | **Items Description** | **Theoretical Support** |
| --- | --- | --- |
|  | **First Fator: Body Acceptance** |  |
| 1 | Do you like your body the way it is? [*Você gosta de seu corpo como ele é?*] | Negy and Winton^1^; Kaplan-Myrth^2^; FG. |
| 2 | Do you try to have a healthy diet? [*Você busca ter uma alimentação saudável?*] | Tylka^3^, Wood-Barcalow, Tylka and Augustus-Horvath^4^; FG. |
| 3 | Do you like your appearance? [*Você gosta de sua aparência?*] | Negy and Winton^1^; Kaplan-Myrth^2^; FG. |
| 11 | Do you seek to be independent? [*Você procura ser independente?*] | FG. |
| 14 | Do you like yourself the way you are? [*Você gosta do jeito que você é?*] | Negy and Winton^1^; Kaplan-Myrth^2^; FG. |
| 15 | Do you take care of your appearance? [*Você cuida de sua aparência?*] | Kaplan-Myrth^2^; FG. |
| 17 | Do you recognize your good qualities? [*Você reconhece suas qualidades?*] | Ceyhan and Ceyhan^5^; Sheerer^6^; FG. |
| 18 | Do you become attentive to your body posture when you are with other people? [*Você fica atento à postura de seu corpo quando está com outras pessoas?*] | Kaplan-Myrth^2^; FG. |
| 19 | Do you think that you are physically attractive? [*Você se considera fisicamente atraente?*] | Negy and Winton^1^; Kaplan-Myrth^2^; FG. |
| 21 | Do You seek to develop your orientation and mobility? [*Você procura desenvolver sua orientação e mobilidade?*] | FG. |
| 28 | Do you practice exercise? [*Você pratica atividade física?*] | Tylka^3^, Wood-Barcalow, Tylka and Augustus-Horvath^4^, Bullington and Karlsson^7^; FG. |
| 29 | Do you believe that your work is worthy? [*Você acredita que seu trabalho tem valor?*] | Sheerer (1949)^6^; FG. |
| 30 | Do you seek to have financial independence? [*Você busca ter independência financeira?*] | FG. |
| 33 | Do you like to take care of you? [*Você gosta de cuidar de você?*] | Negy and Winton^1^; Kaplan-Myrth^2^; FG. |
|  | **Second Factor: Self-protection from social stigmas** |  |
| 8 | Do you avoid persons who judge you by your blindness? [*Você evita pessoas que te julgam pela sua cegueira?*] | Brillhart^8^; Carson and Langer^9^; Sheerer^6^; Tylka^3^, Wood-Barcalow, Tylka and Augustus-Horvath^4^; FG. |
| 13 | Do you worry about some negative societal attitudes about you? [*Você se preocupa com algumas atitudes negativas da sociedade a seu respeito?*] | Brillhart^8^; Carson and Langer^9^; Sheerer^6^; Tylka^3^, Wood-Barcalow, Tylka and Augustus-Horvath^4^; FG. |
| 20 | Do you worry about prejudiced opinions about you? [*Você se preocupa com opiniões preconceituosas a seu respeito?*] | Brillhart^8^; Carson and Langer^9^; Sheerer^6^; Tylka^3^, Wood-Barcalow, Tylka and Augustus-Horvath^4^; FG. |
| 22 | Do you feel worthy, even if you are aware of some sort of prejudice about you? [*Você sente que tem valor, mesmo se sofre algum tipo de preconceito?*] | Brillhart^8^; Carson and Langer^9^; Sheerer^6^; Tylka^3^, Wood-Barcalow, Tylka and Augustus-Horvath^4^; FG. |
| 26 | Do you worry about preposterous questions about yourself? [*Você se incomoda com perguntas inadequadas a seu respeito?*] | Brillhart^8^; Carson and Langer^9^; Sheerer^6^; Tylka^3^, Wood-Barcalow, Tylka and Augustus-Horvath^4^; FG. |
|  | **Third Factor: Feeling and believing in one’s capacities** |  |
| 4 | Do you have good spatial orientation? [*Você possui boa orientação espacial?*] | Kitchin, Jacobson, Golledge and Blades^10^; Sánchez and Torre^11^. |
| 5 | Do you think that you are capable of deciding what is the best for you? [*Você se considera capaz de decidir o que é melhor para você?*] | Kaplan-Myrph^2^. |
| 6 | Do you have a positive attitude towards your life? [*Você tem uma postura positiva em relação a sua vida?*] | Farber^12^. |
| 7 | Do you believe you are able achieve your goals? [*Você acredita que tem condições de alcançar seus objetivos?*] | Farber^12^. |
| 9 | Do you have facilities of relating to other people? [*Você tem facilidades para se relacionar com outras pessoas?*] | James and Stojanovik^13^; Santin and Simmons^14^; FG. |
| 10 | Do you think that your blindness hinder you from taking part in your favorite activities? [*Você acha que sua cegueira lhe atrapalha a fazer coisas que você gosta?*] | Besteiro, Franco, Morales, Sagardoy, and Mateos^15^. |
| 12 | Do you overcome challenges that may exist day-to-day? [*Você supera desafios que possam existir no seu dia-a-dia?*] | Farber^12^ |
| 16 | Do you think being blind makes it difficult to find a romantic partner? [*Você acha que ser cego lhe dificulta encontrar um par para se relacionar amorosamente?*] | Fichten, Goodrick, Amsel and McKenzie^16^. |
| 23 | Do you believe that your opinions are worthless in the places you frequent (work, school, home ...)? [*Você acredita que suas opiniões têm valor nos lugares que você frequenta (trabalho, escola, lar...)?*] | James and Stojanovik^13^, FG. |
| 24 | Do you think that your blindness hinders you from moving to new and unknown places? [*Você acha que sua cegueira lhe dificulta se deslocar para lugares novos e desconhecidos?*] | Kitchin, Jacobson, Golledge and Blades^10^; Sánchez and Torre^11^. |
| 25 | Do you pay attention to different things happening around you? [*Você fica atento a diferentes coisas que acontecem a sua volta?*] | Gougoux, Zatorre, Lassonde, Voss and Lepore^17^, FG. |
| 27 | Do you think that you are able to independently perform tasks in your day-to-day life? [*Você se considera capaz de realizar tarefas de seu dia-a-dia de forma independente?*] | Arruda^18^; Latham and Usherwood^19^. |
| 31 | Can you avoid obstacles on the street? [*Você consegue desviar de obstáculos na rua?*] | Kitchin, Jacobson, Golledge and Blades^10^; Sánchez and Torre^11^ |
| 32 | Are you able to take care of yourself? [*Você se considera capaz de cuidar de você?*] | Arruda^18^, FG. |

FG = Focus Group

Items were rated on a 5-point Likert scale (1 = *never*, 2 = *seldom*, 3 = *sometimes*, 4 = *often*, 5 = *always*)

Brazilian Portuguese original version of the items are given in brackets

**Table S2 -** The item pool refined in Study 2 after content validity (panel of experts and pretest)

| **Items** | **Situation** | **Re^*^** | | **Items Description** |
| --- | --- | --- | --- | --- |
|  |  |  | **First Factor: Body Acceptance** | |
| 1 | Maintained**^*^** | **1** | | Do you like your body the way it is? [*Você gosta de seu corpo como ele é?*] |
| 2 | Changed**^*^** | **8** | | Do you take care of your body seeking to eat healthily? [*Você cuida de seu corpo buscando ter uma alimentação saudável?*] |
| 3 | Changed | **3** | | Do you like your appearance, for example, your hair, your face, the way you dress? [*Você gosta de sua aparência, por exemplo, seu cabelo, seu rosto, seu modo de vestir?*] |
| 11 | Changed | **18** | | Do you strive to be fully independent in your day-to-day life? [*Você se empenha para ser totalmente independente no seu dia-a-dia?*] |
| 14 | Maintained | **14** | | Do you like yourself the way you are? [*Você gosta do jeito que você é?*] |
| 15 | Changed | **15** | | Do you take care of your appearance, for instance, your hair, your skin, and your clothes? [*Você cuida de sua aparência, por exemplo, de seu cabelo, sua pele, seu vestuário?*] |
| 17 | Maintained | **17** | | Do you recognize your good qualities? [*Você reconhece suas qualidades?*] |
| 18 | **Eliminated^*^** |  | |  |
| 19 | Maintained | **19** | | Do you think you are physically attractive? [*Você se considera fisicamente atraente?*] |
| 21 | **Eliminated** |  | |  |
| 28 | Changed | **2** | | Do you take care of your health by practicing physical exercises? [*Você cuida de sua saúde praticando exercícios físicos?*] |
| 29 | Changed | **25** | | Do you give too much value to the activities in which you are currently engaged, eg.: work, study, crafts, among others? [*Você dá muito valor às atividades que você exerce atualmente, por exemplo, trabalho, estudo, artesanato, entre outras?*] |
| 30 | **Eliminated** |  | |  |
| 33 | Changed | **4** | | Do you strive to be fully independent in locomotion? [*Você se empenha para ser totalmente independente na sua locomoção?*] |
|  |  |  | | **Second Factor: Self-protection from social stigmas** |
| 8 | **Eliminated** |  | |  |
| 13 | Changed | **13** | | Do you worry about negative attitudes from society regarding your blindness? [*Você se incomoda com algumas atitudes negativas da sociedade a respeito de sua condição de cego?*] |
| 20 | Changed | **20** | | Do you get annoyed with prejudiced opinions of your blindness? [*Você se incomoda com opiniões preconceituosas a respeito de sua cegueira?*] |
| 22 | Changed | **22** | | When you observe some kind of prejudice related with your blindness, do you feel yourself to be less of a person? [*Quando você observa algum tipo de preconceito social em relação a sua cegueira, você se sente inferior às outras pessoas?*] |
| 26 | Changed | **26** | | Do you worry about preposterous questions about your blindness? [*Você se incomoda com perguntas inadequadas sobre sua cegueira?*] |
|  |  |  | | **Third Factor: Feeling and believing in one’s capacities** |
| 4 | **Eliminated** |  | |  |
| 5 | Maintained | **5** | | Do you think that you are capable of deciding what is best for you? [*Você se considera capaz de decidir o que é melhor para você?*] |
| 6 | Changed | **6** | | Are you highly positive about your life? [*Você é muito positivo em relação a sua vida?*] |
| 7 | **Eliminated** |  | |  |
| 9 | Changed | **9** | | Does blindness cause difficulties in your social interactions? [*Ser cego lhe dificulta se relacionar com outras pessoas?*] |
| 10 | Changed | **10** | | Does blindness hinder you from taking part in your favorite activities? [*Sua cegueira lhe atrapalha a fazer coisas que você gosta?*] |
| 12 | Changed | **12** | | Do you feel that you are capable of overcoming your day-to-day difficulties? [*Você se sente capaz de superar as dificuldades que possam existir no seu dia-a-dia?*] |
| 16 | Changed | **16** | | Does being blind have negative effect on your romantic relationships? [*Ser cego lhe dificulta ter um relacionamento amoroso?*] |
| 23 | Changed | **23** | | Are your opinions highly respected in the places you give them, for example, in your work, school, and home? [*Suas opiniões são muito respeitadas nos lugares que você frequenta, por exemplo, trabalho, escola, lar?*] |
| 24 | Changed | **11** | | Do you have the capability to move into new and unknown environments? [*Você tem facilidade para se deslocar em ambientes novos e desconhecidos?*] |
| 25 | Changed | **21** | | Do you connect with everything that happens around you, for example, noises, sounds, and smells? [*Você fica ligado em tudo que acontece a sua volta, por exemplo, ruídos, sons, cheiros?*] |
| 27 | Changed | **7** | | Do you think that you are able to perform day-to-day tasks independently, for example, cooking, shopping, and choosing clothes, among others? [*Você se considera capaz de realizar tarefas de seu dia-a-dia de forma independente, por exemplo, cozinhar, fazer compras, escolher roupas, entre outras?*] |
| 31 | **Eliminated** |  | |  |
| 32 | Changed | **27** | | Do you think that you are able to care of your appearance, for example, put on makeup or shave, arms or legs, match clothes? [*Você se considera capaz de cuidar de sua aparência, por exemplo, fazer maquiagem ou barba, depilar braço ou perna, combinar roupas?*] |
|  | Suggested new item | **24** | | Does blindness hinder you from doing the things you have to do? [*Sua cegueira lhe atrapalha a fazer coisas que você necessita fazer?*] |

**^*^**Re= renumbered – items have been renumbered as suggested experts, so that the simplest stay at the beginning of the scale.

**^*^**Maintained - Item was retained as in the original version

**^*^**Changed - Item suffered minor or major changes to get content validity

**^*^Eliminated –** Item was dropped because it did not obtain content validity

Brazilian Portuguese original version of the items are given in brackets

**References SI:**

1. Negy C, Winton S (2008) A comparison of pro- and anti-nudity college students on acceptance of self and of culturally diverse others. J Sex Res 45: 287-294.
2. Kaplan-Myrth N (2000) Alice without a looking glass: blind people and body image. Anthropol Med 7: 277-299.
3. Tylka TL (2011) Positive Psychology perspectives on body image. In: Cash, TF.; Smolak, L. Body image: a handbook of science, practice, and prevention. New York: Guilford press.
4. Wood-Barcalow NL, Tylka TL, Augustus-Horvath CL (2010) ‘‘But I Like My Body’’: Positive body image characteristics and a holistic model for young-adult women. Body Image 7: 106-116.
5. Ceyhan A, Ceyhan E (2011) Investigation of university students’ self-acceptance and learned resourcefulness: a longitudinal study. High Educ 61: 649-661.
6. Sheerer ET (1949) An analysis of the relationship between acceptance of and respect for self and acceptance of and respect for others in ten counseling cases. J Consult Psychol 13: 169-175.
7. Bullington J, Karlsson G (1997) Body experiences of persons who are congenitally blind: a phenomenological-psychological study. J Child Psychol Psyc 91: 151-162.
8. Brillhart B (1986) Predictors of self-acceptance. Rehabil Nurs 11: 8-12.
9. Carson SH, Langer EJ (2006) Mindfulness and self-acceptance. J Ration Emot Cogn Behav Ther 24: 29-43.
10. Kitchin, RM, Jacobson RD, Golledge RG, Blades M (1998) Belfast without sight: exploring geographies of blindness. Irish Geography 31: 34-46.
11. Sánchez J, Torre N (2010) [Autonomous navigation through the city for the blind](http://www.scopus.com.ez25.periodicos.capes.gov.br/record/display.url?eid=2-s2.0-78650617943&origin=resultslist&sort=plf-f&src=s&st1=Autonomous+navigation+through+the+city+for+the+blind&sid=w_XCGotapo1fLiMnYVRsR3L%3a250&sot=q&sdt=b&sl=72&s=TITLE-ABS-KEY-AUTH%28Autonomous+navigation+through+the+city+for+the+blind%29&relpos=0&relpos=0&searchTerm=TITLE-ABS-KEY-AUTH%28Autonomous%20navigation%20through%20the%20city%20for%20the%20blind%29). ASSETS'10 - Proceedings of the 12th International ACM SIGACCESS Conference on Computers and Accessibility, 30: 195-202.
12. Farber RS (2000) Mothers with disabilities: in their own voice. Am J Occup Ther 54: 260–268.
13. James DM, Stojanovik V (2006) Communication skills in blind children: a preliminary investigation. Child Care Health Dev 33: 4–10.
14. Santin S, Simmons JN (1977) Problems in the construction of reality in congenitally blind children. J Visual Impair Blin 71: 425 – 429.
15. Besteiro MPV, Franco CP, Morales, LG, Sagardoy, RC, Mateos, AGL (2009) La razón y la emoción: integración de las intervenciones cognitivoconductuales y experenciales en el tratamiento de los trastornos de alimentación de larga evolución. Nutr Hosp 24: 614-617.
16. Fichten CS, Goodrick G, Amsel R, McKenzie SWl (1991) Reactions toward dating peers with visual impairments. Rehabil Psychol 36: 163-178
17. Gougoux F, Zatorre RJ, Lassonde M, Voss P, Lepore F (2005) A functional neuroimaging study of sound localization: visual cortex activity predicts performance in early-blind individuals. PLoS Biol 3: 324-333.
18. Arruda SMCP (2006) Percepções da auto-eficácia nas atividades de vida diária e qualidade de vida de estudantes com baixa visão ou cegueira [Self-efficacy perceptions in daily activities and life quality for students with low vision or blindness]*.* [tese]. Campinas: Faculdade de Ciências Médicas da Universidade Estadual de Campinas.
19. Latham K, Usherwood C (2010) Assessing visual activities of daily living in the visually impaired. Ophthalmic and Physiological Optics 30: 55-65.
